# Systematic Review of Clinical Insights into Novel Coronavirus (CoVID-19) Pandemic: Persisting Challenges in U.S. Rural Population

**DOI:** 10.3390/ijerph17124279

**Published:** 2020-06-15

**Authors:** Hari Vishal Lakhani, Sneha S. Pillai, Mishghan Zehra, Ishita Sharma, Komal Sodhi

**Affiliations:** Departments of Surgery and Biomedical Sciences, Marshall University Joan C. Edwards School of Medicine, Huntington, WV 25755, USA; lakhani@marshall.edu (H.V.L.); pillais@marshall.edu (S.S.P.); humayun@marshall.edu (M.Z.); sharma11@marshall.edu (I.S.)

**Keywords:** coronavirus, pandemic, clinical characteristics, pharmacotherapies, rural healthcare

## Abstract

(1) Introduction. A recent viral outbreak of novel coronavirus (CoVID-19) was declared as a pandemic by the World Health Organization (WHO) due to its global public health concern. There has been an aggressive growth in the number of emerging cases suggesting rapid spread of the virus. Since the first reported case of CoVID-19, there has been vast progress in understanding the dynamics of CoVID-19. However, there is an increasing evidence of epidemiological disparity in disease burden between urban and rural areas, with rural areas having minimal pandemic preparedness and their own healthcare challenges. Therefore, this review aims to provide insight on the pathogenesis and the transmission dynamics of CoVID-19 along with pharmacological and non-pharmacological intervention strategies to mitigate the clinical manifestation of this virus. This review also aims to assess existing challenges of the CoVID-19 pandemic in rural areas based on past pandemic experiences and the effect on rural population. (2) Methods. A literature review was conducted using databases such as PubMed, Science Direct, Academic Search Premier, ProQuest, and Google Scholar, along with information from governmental organizations such as Centers for Disease Control and Prevention (CDC) and World Health Organization (WHO). (3) Results. The causative virus, with its likely zoonotic origin, has demonstrated high pathogenicity in humans through increasing human-to-human transmission leading to extensive mitigation strategies, including patient quarantine and mass “social distancing” measures. Although the clinical manifestation of symptoms is mild in majority of the virus-inflicted population, critical patients may present with pneumonia and acute respiratory distress syndrome, exacerbated by pre-existing comorbidities, eventually leading to death. While effective coronavirus disease (CoVID-19)-specific vaccines and drugs are under clinical trials, several pharmacological and non-pharmacological interventions have been adapted to manage symptoms and curtail the effect of the virus to prevent increasing morbidity and mortality. Several persisting challenges have been noted for mitigating CoVID-19 in rural areas, including the poor healthcare infrastructure, health literacy, pandemic preparedness along with the fact that majority of rural population are frail subjects with pre-existing comorbidities. (4) Discussion. The increasing rate of incidence of CoVID-19 presents its own challenges, burdening healthcare institutions and the global economy, and impacting the physical and mental health of people worldwide. Given the clinical insights into CoVID-19 and the challenges presented in this review for the U.S. rural population, mitigation strategies should be designed accordingly to minimize the morbidity and mortality of this contagion.

## 1. Introduction

The outbreak of novel coronavirus, severe acute respiratory syndrome coronavirus (SARS-CoV-2), first reported in Wuhan, China, that contributed to an increased morbidity and mortality, was declared to be a major worldwide pandemic by the World Health Organization (WHO) [1]. Since then, recent advances in understanding the pathological progression and transmission of coronavirus disease (CoVID-19) have contributed to efforts toward the development of pharmacological and non-pharmacological strategies. Although, coronaviruses (CoVs) were initially contemplated to be a cause of multifaceted diseases in mammals and birds with its origin in bats [2], the evolution of this virus has resulted in its increased pathogenicity in humans. CoVs, in general, were deemed important in veterinary responses; however, since the outbreak of human coronavirus (HCoV) causing severe acute respiratory syndrome (SARS-CoV) in 2003, Middle East respiratory syndrome coronavirus (MERS-CoV) in 2012, and the recent outbreak of SARS-CoV-2, it has been labeled as an “emerging pathogen” in humans.

Given their high pathogenicity in humans and the extent of global impact caused by the HCoV outbreaks in the past two decades, it has been a topic of great interest; hence, the mechanistic action of this virus is being thoroughly investigated by researchers. The causative agent of the current pandemic presenting as a viral pneumonia, SARS-CoV-2 is a group of large, enveloped RNA viruses under the Coronaviridae family, classified specifically under the Nidovirales order [3]. The cumulative line of evidence demonstrates that SARS-CoV-2 shares 79.6% of sequence identity to the SARS-CoV outbreak in 2003, at a genomic level, while the genomic sequence is almost 96% identical to the bat coronavirus [4], which provides a significant insight to understanding CoVID-19 and its origin. While understanding the origin and the evolution of SARS-CoV-2 is imminent, the clinical manifestation of this virus in humans and the increasing number of symptomatic and asymptomatic “carriers” every day, presents growing concern for public health.

The aggressive growth in numbers of emerging cases suggests the rapid spread of the virus in naïve population and improvement in diagnostic capability during this CoVID-19 outbreak, however, there is an increasing evidence of epidemiological disparity in disease burden between urban and rural areas [5,6,7]. The differences in the urban and rural areas can highly determine the influence of a viral pandemic, in terms of viral transmission, diagnostics, morbidity, and mortality [5]. These differences mainly arise from the socioeconomic factors, access to healthcare, and pandemic preparedness. Given the higher percentage of U.S. rural population being old (age ≥ 60), individuals with obesity (body mass index (BMI) ≥ 30) and associated co-morbidities along with their smoking status; these factors puts them at higher risk of infection and severe complications arising from CoVID-19. Hence, this review aims to provide an insight on the modality and transmission dynamics of the SARS-CoV-2 clinical characteristics persistent with the virus and further discuss efforts toward the development of pharmacological and non-pharmacological strategies to combat its progression in humans. This review also aims to assess the challenges of the CoVID-19 pandemic in rural settings based on the past pandemic experiences and the overall effect on rural population.

## 2. Methods

The methodology of this study followed the principles of literature review. The study was conducted based on literature identification and collection, literature analysis, and literature categorization.

### 2.1. Literature Identification and Collection

The databases of this research included Academic Search Premier, PubMed, ProQuest, Science Direct, and Google Scholar. An extensive search was performed from various governmental websites including the World Health Organization (WHO) and Centers for Disease Control and Prevention (CDC). The keywords used for the search included the following: “coronavirus” OR “pandemic” OR “pathogenesis” OR “SARS-CoV-2” AND “CoVID-19” OR “human transmission” OR “pharmacological” AND “nonpharmacological” OR “clinical characteristics” AND “the U.S.” AND “rural healthcare”. The articles were retrieved from our search and further distinguished for relevancy.

### 2.2. Literature Analysis

The research articles chosen were reviewed for government guidelines, mitigation strategies, persisting symptoms, CoVID-19 morbidity and mortality, and U.S. rural healthcare. The inclusion and exclusion criteria were set to only evaluate articles and data published from 2003 to 2020 in order to limit this search. Furthermore, this search was restricted to all the articles available as full text and published in the English language. Any relevancy or information deemed important was also included from articles published outside U.S. A total of 181 articles was assessed by three reviewers for relevancy, out of which 96 references and citations were utilized.

### 2.3. Literature Categorization

The articles were reviewed based on their abstracts to find out the relevancy to this study. Conclusively, the findings were presented under the subheadings of: Pathogenesis of CoVID-19, Transmission Dynamics of CoVID-19, Clinical Characteristics Persistent with CoVID-19, Pharmacological and Non-pharmacological Intervention, and, finally, Challenges in Rural Population.

## 3. Results

### 3.1. Pathogenesis of CoVID-19

CoVs normally cause enteric diseases in several animal species and affect the respiratory tract of mammals, including humans, and lead to mild-to-severe respiratory tract infections [8]. Studies report five major protein regions for HCoVs, such as replicase complex, spike, envelope, membrane, and nucleocapsid proteins, that function in viral structure assembly and replications [9]. The overlapping open reading frames (ORFs) of replicase complex, *ORF1a* and *ORF1b*, encode 16 non-structural proteins (nsp) of viral RNA synthesis complex through proteolytic processing [10]. The *nsp12* is a viral RNA-dependent RNA polymerase, together with co-factors *nsp7* and *nsp8* possessing high polymerase activity. A recent study showed 94.4% similarity in the amino acid sequences of the seven conserved replicase domains in ORF1ab of SARS-CoV and SARS-CoV-2 [4]. The spike (S) is a transmembrane glycoprotein, that comprises two domains: S1 and S2, which play a pivotal role in mediating viral infection through binding the host receptor [11,12]. The interaction between the S1 domain and its cognate receptor triggers a conformational change in the S protein, which then promotes membrane fusion between the viral and cell membrane through the S2 domain [13]. The three short insertions in the N-terminal domain as well as changes in four out of five of the key residues in the receptor-binding motif compared with the sequence of SARS-CoV are the key differences recently found by scientists in the *S* gene of SARS-CoV-2 [4]. The major host cell receptors utilized by all HCoVs are aminopeptidase N by HCoV-229E [14], angiotensin-converting enzyme 2 (ACE2) by SARS-CoV [15] and HCoV-NL63 [16,17], dipeptidyl peptidase 4 (DPP4) by MERS-CoV [18], and 9-O-acetylated sialic acid by HCoV-OC43 and HCoV-HKU1 [19,20].

A recent study showed that CoVID-19 is able to utilize the same membrane-bound ACE2 as an entry receptor in ACE2-expressing cells with higher affinity than SARS-CoV and does not use other coronavirus receptors [4,21]. ACE2 is highly expressed in the mouth and tongue, facilitating viral entry in the host, and in lungs it is expressed in lower lungs on type I and II alveolar epithelial cells. After infection, SARS-CoV-2 entry starts with the binding of the spike glycoprotein expressed on the viral envelope to ACE2 on the alveolar surface. The binding of SARS-CoV-2 to ACE2 stimulates the clathrin-dependent endocytosis of the whole SARS-CoV-2 and ACE2 complex, inducing fusion at the cell membrane [22]. The membrane fusion and endosomal cell entry is facilitated by the low pH in the cellular environment and pH-dependent endosomal cysteine protease cathepsins [8]. Once inside the cells, SARS-CoV-2 exploits the endogenous transcriptional machinery of alveolar cells to replicate itself and spreads through the entire lung [8]. The nucleocapsid helps for packaging the viral genome through protein oligomerization. As the infection progresses, it deleteriously affects the normal activity of most of the ciliated cells in the alveoli that clear the airways, with a consequent progressive accumulation of debris and fluids in the lungs and eventually leads to acute respiratory distress syndrome (ARDS). In addition to the alveolar cells in the lungs, ACE2 expression has been reported in other organs, including the kidney, the heart, and the gut [23], which supports the commonly reported co-morbidities of CoVID-19, such as acute kidney injury (AKI), cardiac damage, and abdominal pain.

### 3.2. Transmission Dynamics of CoVID-19

As the first cases of the CoVID-19 were linked to direct exposure to the Huanan Seafood Wholesale Market of Wuhan, China, animal-to-human transmission was initially presumed [24,25]. However, subsequent cases revealed the scope of human-to-human transmission through the individuals in the incubation stage or showing symptoms. Through the widespread information available and recent increase in understanding of the SARS-CoV-2, the virus appears to have an incubation time ranging from 2 to 14 days [26,27,28,29]. A recent report suggested that the symptomatic patients had higher viral loads, detected in the nose and throat, following the onset of their symptoms [30]. These viral loads peaked around 5–6 days following the symptoms onset [31]. Consequently, the asymptomatic patients had a viral load similar to symptomatic patients [30]. Hence, such human-to-human transmission through symptomatic and asymptomatic carriers caused the epidemic to gradually grow over weeks, eventually becoming a global pandemic. The human-to-human transmission is mainly through the inhalation of respiratory droplets from coughing and sneezing and through contact of infected surfaces, then mediating the infection through the mouth, nose, or eyes [32]. Transmission via the inhalation of exhaled respiratory droplets may occur as aerosol droplets that can survive for prolonged periods, mediating long-range human-to-human transmission via air movement. Inhalation of virus-laden fine particles could transport the virus into deeper alveolar and tracheobronchial regions, which could increase the chance of infective transmission and oxidant pollutants in air can impair the immune function and attenuate the efficiency of the lung to clear the virus in the lungs. Pro-inflammation, injury, and fibrosis from inhaled airborne particulate matters combined with an immune response or cytokine storm induced by SARS-CoV-2 infection could enhance the infection severity [32]. Since human-to-human transmission is the primary mode of transmission of the virus, the international public health response toward mitigating CoVID-19 has largely been based on social/physical distancing, isolation of cases, and quarantine measures [25].

Further studies have also shown that infectious viruses, including CoVs, can survive for long periods outside of the host organism, increasing the opportunity for transmission via touch [33]. It has been reported that SARS-CoV-2 can last about three days on plastic and stainless steel surfaces, about one day on cardboard surfaces, and about four hours on copper surfaces [34,35]. Despite these evidences, this is not thought to be the main way the virus spreads (CDC). Apart from that, CoVs were previously reported to remain infectious in water and sewage for days to weeks [36], which adds another potential transmission route if the quality of personal hygiene is poor. However, the mode of transmission for CoVID-19 through contaminated water does not have enough scientific evidence, hence, requires further studies to validate it.

### 3.3. Clinical Characteristics Persistent with CoVID-19

Among the earlier studies, CoVID-19 was reported to have a mean serial interval of 7.5 days, which is defined as the delay between disease onset dates in successive cases in chains of transmission [37]. Using the data from the serial interval distribution, the basic reproductive number was estimated to be 2.2 [37]. The identification of these characteristics is important as the study extensively inferred the doubling of CoVID-19 every seven days since each patient transmitted the infection to an additional 2.2 individuals. Similarly, other modeling studies estimated the basic case reproduction rate ranging from 2 to 6.47, while WHO published their early estimation to be ranging from 1.4 to 2.5 [38,39,40,41].

CoVID-19 may present with a range of symptoms in humans, varying from mild cold-like symptoms to severe life-threatening respiratory tract infections. The infection begins with the gradual onset of symptoms following an incubation period of 2–14 days after exposure [26,27,28,29,42]. On the basis of disease severity, clinical manifestations can be categorized as mild (81%), severe (14%), and critical (5%), as noted in the CoVID-19 positive patients [41]. The initial infectious stage is characterized by mild constitutional symptoms and upper respiratory tract infection [41]. The most frequently experienced symptoms include fever, dry cough, sore throat, sputum production, fatigue, shortness of breath, and headache [42]. This is followed by other vague symptoms such as nasal congestion, rhinorrhea, sneezing, malaise, muscle pain, drowsiness, and myalgia [29,41,43,44,45]. With the number of cases increasing worldwide, other symptoms have also been reported, which are ever evolving and differ depending on the population studies. However, the question whether these symptoms are directly correlated with CoVID-19 manifestation, remains elusive and requires further scientific and clinical evidence.

Following an antecedent of 5–9 days of primary viral manifestations, these initially mild symptoms manifest as a progressively advanced disease accompanied by pneumonia and ARDS, which is the most ominous feature of this infection [29,41,43,46]. Other secondary manifestations, which could be present, are RNAemia as a continuum of ARDS, chronic dyspnea, tachypnea, hypoxia, diarrhea, abdominal pain, metabolic acidosis, and coagulation dysfunction [41,42,47,48]. Moreover, in critical cases, the virus can also trigger severe complications such as sepsis and septic shock, multiple organ dysfunction, and acute cardiac and renal injury [29,41,42,43]. These complications are also linked with high levels of pro-inflammatory cytokines [29,49] and hematological changes including leukopenia (33.7%), lymphopenia (83.2%), and thrombocytopenia (36.2%) [49]. Critical cases with secondary infections may also present co-infections of bacteria and fungi [43].

In addition to these overt symptoms, there has been a growing concern for asymptomatic cases [50]. These immunocompetent viral carriers present asymptomatic infections, not manifesting any symptoms, which unanimously pose an imminent danger and may have grave outcomes. Recent studies have signified the emergence of asymptomatic or mildly symptomatic infections as a potential portal of viral transmission that needs vigilant control to detect and mitigate these community-acquired infections [37,46,51,52]. Individuals with viral tolerance present a potential challenge in the annihilation of this infection, especially in rural America, with inadequate health care infrastructure and high rates of chronic clinical issues [53]. Consistent with recent studies, the majority of critically ill CoVID-19 patients are known to have some underlying chronic medical conditions such as cardiovascular disease, diabetes, and chronic respiratory disease [29,41,42,45]. These clinical conditions are also a significant health issue in the rural population; therefore, such patients are more vulnerable to developing severe CoVID-19 symptoms [54,55,56,57,58,59].

### 3.4. Pharmacological Interventions

As there are no specific antiviral drugs or vaccine recommended specifically for SARS-CoV-2 infection for potential therapy of humans till date, scientists are endeavoring to find drugs to treat this disease. Currently, the treatment is symptomatic and oxygen therapy is considered as the major treatment intervention for patients with severe infection [41]. Pharmacological interventions that can prevent a mild state from progressing to the severe or critical state will significantly improve the overall prognosis of the disease. In order to reduce disease worsening and the mortality rate, developing an effective approach to modulate the immune system or suppress reactive cytokine production is of crucial importance. Studies are currently testing the efficacy of existing broad-spectrum antiviral drugs in inhibiting SARS-CoV-2 replication. The major drugs that exhibited promising inhibitory effects are remdesivir and chloroquine. Remdesivir is an adenosine analogue that cause premature termination of viral replication and has been recently recognized as a promising antiviral drug against a wide array of RNA virus (including SARS/MERS-CoV5) infections in various experimental models [60,61]. Reports show that remdesivir can inhibit CoVID-19 infection efficiently in a human cell line (human liver cancer Huh-7 cells), which is sensitive to CoVID-19 [39]. Recent studies have also reported that remdesivir yielded promising results in the treatment of a patient with CoVID-19 in the U.S. [46], and further studies are currently under progress to evaluate the efficacy and safety of the drug. Chloroquine is an antimalarial drug, known to exert a potent antiviral effect by virtue of its ability to increase the endosomal pH required for virus/cell fusion and interfere with the glycosylation of cellular receptors of SARS-CoV [62]. A study demonstrated that chloroquine could function at both, entry and at post-entry stages of the CoVID-19 infection in Vero E6 cells and has an immune-modulating activity, which may synergistically enhance its antiviral effect in vivo [39]. Hydroxychloroquine is an antirheumatic drug with a similar chemical structure to chloroquine and exhibits a strong immunomodulatory capacity, which prevents inflammation flare-ups and organ damage [63]. It can also increase the intracellular pH, interrupt toll-like receptor (*TLR*) signaling and subsequent attenuation of proinflammatory signaling activation and production of cytokines, such as type I interferons, interleukin-1 (*IL-1*), and tumor necrosis factor-α (*TNF-α*) [64]. As severe CoVID-19 can be due to the overactivation of the immune system triggered by CoVID-19 infection, hydroxychloroquine can be suggested to attenuate the progression of the disease from mild to severe. IFN-α is a broad-spectrum antiviral used to treat hepatitis and is reported to inhibit SARS-CoV reproduction in vitro [65]. Lopinavir/ritonavir, a medication used to treat the human immunodeficiency virus (HIV), is also found to exhibit anti-SARS-CoV activity in vitro and in clinical studies [66]. Ribavirin, a nucleoside analogue with a broad-spectrum of antiviral effects, exhibited lower risk of ARDS and death when used in combination with lopinavir/ritonavir [65]. Favipiravir is a new type of RNA-dependent RNA polymerase inhibitor reported to possess anti-influenza virus activity and is capable of blocking the replication of flavi-, alpha-, filo-, bunya-, arena-, noro-, and other RNA viruses [67]. So clinical trials of favipiravir on CoVID-19 infection have now been initiated by the Clinical Medical Research Center of the National Infectious Diseases and the Third People’s Hospital of Shenzhen. Other potential drugs include type II transmembrane serine protease (TMSPSS2) inhibitors and BCR-ABL kinase inhibitor imatinib. The ability of these drugs to block the entry and inhibit the fusion of virions with the endosomal membrane may provide anti-coronal activity [68,69].

As discussed earlier in the light of recently published reports, 2019-nCoV can utilize ACE2 as an entry receptor in ACE2-expressing cells with higher affinity than SARS-CoV, suggesting potential drug targets for therapeutic development [4]. ACE2 also plays an important role in the renin-angiotensin system (RAS), and the imbalance between ACE/Ang II/AT1R pathway and ACE2/Ang (1–7)/Mas receptor pathway in the RAS will lead to multisystem inflammation, and studies have shown that RAS inhibitors could effectively relieve symptoms of acute severe pneumonia and respiratory failure [70]. One of our previously published articles highlights the possible interactions between a cytochrome P450 (CYP)-derived arachidonic acid metabolite, 20-hydroxyeicosatetraenoic acid (20-HETE), and RAS [71]. The findings showed that 20-HETE-induced hypertension in CYP4A2-transduced rats is associated with RAS upregulation and can be abrogated by ACE inhibition or angiotensin type 1 receptor (AT1R) blockade. The study evaluates the effect of lisinopril, losartan, 20-HEDE (20-HETE antagonist 20-hydroxyeicosa-6(Z), 15(Z)-dienoic acid), and HET0016, an inhibitor of 20-HETE, in modulating RAS, ACE, and various downstream signaling molecules involved in the pathway. Hence, our findings will also provide some valuable lead for the ongoing and future investigations on therapeutic interventions against CoVID-19. As the pandemic continues to affect increasing an number of people, scientists around the world are actively exploring the efficacy and safety of the drugs in the treatment of CoVID-19 and the findings need to be validated in further preclinical and clinical trials.

### 3.5. Non-Pharmacological Interventions

Non-pharmaceutical public health measures are vital in curtailing disease spread [41,45,72]. To constrain local transmission and to reduce the impact of the CoVID-19 pandemic, the WHO has recommended non-pharmacological interventions that are specifically geared to limit the spread of the infection from person to person and reduce the number of mortalities [41]. Strategies to mitigate the transmission entail personal and community-based actions [29]. Non-pharmacological interventions are perhaps some of the most effective ways to prevent widespread mortality of the disease by slowing the rate of infection to a manageable level that healthcare systems can withhold.

Personal interventions include, frequent hand washing after every 15–20 min [29], use of hand sanitizers with ≥60% alcohol content, wearing face masks, encouraging people to cover their mouth and nose during coughing and sneezing, keeping a distance from sick people, avoiding contact with face and mouth with unwashed hands [41,45], and self-quarantine when a person feels unwell [41]. Whereas, community-based mitigation strategies include, social distancing, avoiding crowded areas, and restriction on public gathering and non-essential traveling [29,41].

In addition to these measures, studies have shown the supportive effects of herbal or nutritional interventions, including immune-boosting nutrients and herbs that can help overcome upper respiratory tract viral infections [73]. Complementary therapies that can boost the immune response at the initial stage of CoVID-19 infection might be a prudent approach to overcome the symptoms associated with this infection [74]. There has also been evidence that traditional or herbal medicine has helped improve the condition of patients with CoVID-19 or those who presented with symptoms. A study in the Chinese population following traditional Chinese medicine found that patients who were treated with qingfei paidu decoction (QPD), a herbal Chinese traditional medicine (a combination of 4 different herbal formulas consisting of 21 herbs in total), were found to have an effective cure rate of over 90% against 701 confirmed cases of CoVID-19 [75,76]. In light of this evidence, the Chinese government promoted this herbal treatment to be used in the diagnosis and treatment of patients who have or are suspected to have CoVID-19. QPD was also recommended by the Korean government’s guidelines for severe cases of CoVID-19 [76]. Combined remedies of both herbal medicine and western medicine were also found to be more effective than using western medicine alone in treating patients with CoVID-19 [77].

Furthermore, in response to the current pandemic, WHO recently supported the use of scientifically proven traditional medicines. The recognition by WHO toward traditional, herbal, and complementary medicine, included the use of the medicinal plant *Artemisia annua* against coronavirus, which is being used by African countries. The use of *Artemisia annua* was also boosted by Madagascar in the form of an herbal drink, widely known as “Covid-Organics”. Although there is ample evidence present for the positive effects of this medicinal plant [78,79,80,81], WHO advised testing the efficacy and adverse side effects of this plant through rigorous clinical trials, before prescribing it to CoVID-19 affected patients. Therefore, non-pharmacological interventions suggested by WHO and used by other countries possess potential defense against the spread of this infection, even when drugs and vaccines will be available. These measures can provide protection to the individuals exposed to the virus, by limiting the symptomatic complications arising from CoVID-19, eventually reducing the overall mortality rate. Future studies are recommended to provide valid and reliable containment measures to control the local transmission of this infection.

### 3.6. Challenges in Rural Population

The worldwide escalation and clinical manifestation of CoVID-19, despite constant efforts from the healthcare workers and respective governments to minimize its impact, may still be underdiagnosed. Studies have demonstrated that the risk of mortality from CoVID-19 is significantly higher in the aging population, including patients with pre-existing comorbidities. Reflecting on earlier data, 80% of the mortality related to CoVID-19 was noted among adults aged ≥60 years [82]. These observations raise serious public health concerns, considering that 19.3% of the U.S. population resides in rural areas with the average age of rural Americans being 73.3 years [83] (U.S. Consensus). There is also a high rate of obesity in rural populations [54]. This is important as there are indications that a possible reason for a higher mortality rate for CoVID-19 in Italy compared to China could be the fact that there is a higher percentage of obesity in older adults in Italy, noting a similar pattern that occurred with the H1N1 pandemic [84]. Besides this, rural areas tend to have a higher percentage of smoking within the population [85]. CoVID-19 being a respiratory illness, individuals with a smoking history have higher chances of being affected by the virus and having a severe case that requires ventilation is compounded. While several strategies have been adopted to prevent the spread in urban populations, preventing the transmission dynamics and the spread of CoVID-19 in rural regions have received limited attention. Although rural regions have limited human mobility and relatively lower population densities than urban settings [86], the extent of morbidity and mortality might be significantly higher in the viral-inflicted rural population, making the containment and mitigation of viral transmission unmanageable.

Apart from the potential significant impact of CoVID-19 on the aging population in rural America, the rural healthcare delivery system remains relatively under-resourced. Rural areas across the world have been greatly impacted by pandemics in the past, and it is clear with CoVID-19 that there are on-going issues related to the infrastructure in these communities that have not been addressed. Previous pandemics such as the H1N1, Influenza A caused high mortality rates in rural Turkey, and these deaths were directly linked to lack of advanced intensive care unit (ICU) facilities with preliminary ventilator support [87]. This could easily contribute to the existing problem among other issues such as late diagnosis and late antiviral therapy. The limited availability of ICU facilities is still an issue in rural areas, particularly in the United States. Although there has been a trend of an increasing number of ICU beds being added to hospitals, these ICU beds have been found to be concentrated in large, urban hospitals while small-to-medium hospitals had decreased ICU capacity [88]. As noted previously, severe cases of CoVID-19 can require ventilation, which is primarily available in ICUs. With rural populations having less access to these resources, the mortality rate can rise. Besides a lack of ICU availability, the healthcare in rural America has noted a significant disparity in terms of the distribution of professional resources, such as lack of healthcare workers, major healthcare institutions, and overwhelming financial burden, as compared to urban settings [89]. Limited physicians, healthcare training opportunities, delayed care, disparities in patient demographics (older population), and substandard quality of healthcare are already reflected in overall health outcomes as the mortality is higher in rural populations [90]. These observations suggest lack of pandemic preparedness in rural America; hence, the global spread of CoVID-19 may significantly impact rural communities to a greater extent.

The effectiveness of CoVID-19 pandemic mitigation primarily requires a high level of participation from each individual, such as having adequate knowledge of the viral transmission, engaging in appropriate self-care, following self-hygiene guidelines and social distancing, which are fundamental in avoiding preventable hospitalizations [29]. However, due to lower socioeconomic resources in a rural setting, there is limited literacy, specially health literacy, among the rural population, which may exacerbate the impact of CoVID-19 in rural areas [91]. The cumulative line of evidence have demonstrated that poor adult literacy is strongly correlated with reduced health knowledge and self-management skills, reduced physical and mental health, higher rates of hospitalizations and increased morbidity and mortality [92,93,94]. Hence, the consequences of CoVID-19 pandemic for the rural population will depend partly on the health risk communication and viral awareness. Pandemic communication gaps may increase vulnerability among rural populations, which may trigger disease misconceptions, uninformed policies, insufficient planning, and fostering non-resilience, predisposing the population to communicable viral risks [95].

One way to potentially fix this gap could be by using and ensuring that the local news media (i.e., local news channels, local newspapers) have accurate, current, and practical advice for their viewers. Rural areas rely heavily on media specifically, local media, for their information. Yet a study on a rural area in California found that in general, many local news sources did not include important information about health issues that were affecting the area [96]. While dealing with CoVID-19, it is imperative we use these key news sources to reach areas that traditional media will not. If accurate data and advice can be presented in these sources, it can be an indispensable asset in preventing the reach of inaccurate misconceptions and increase participation in community-based mitigation strategies.

## 4. Discussion

**Figure 1:**
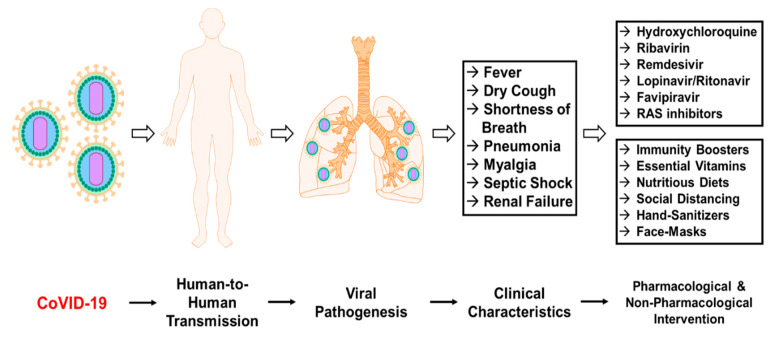
Schematic representation for pathogenicity of novel coronavirus disease (CoVID-19) in humans. The viral transmission through human-to-human induces systemic effects and targets lungs causing clinical manifestation of symptoms associated with the virus that have been persistent in patients affected with CoVID-19. The pharmacological and non-pharmacological intervention strategies may mitigate these effects and assist in viral containment.

The viral outbreak of CoVID-19, declared as a global pandemic, continues to overwhelm the healthcare system with increasing number of patients presenting with clinical symptoms of this coronavirus. This increasing rate of incidence of this contagion presents with its own challenges, burdening healthcare institutions, global economy, impacting the physical and mental health of people worldwide. Although several strategies have been adopted to mitigate the effects of this viral outbreak, the rural population has been largely ignored, despite the high risks of morbidity and mortality among this population. This review aims to provide a clinical insight into the outbreak of CoVID-19, outlining the pathogenesis, transmission dynamics, and clinical characteristics of the patients tested positive for this virus (Figure 1). Although the development of a SARS-CoV-2-specific vaccines is still under clinical trials, this review discusses pharmacological and non-pharmacological interventions that are widely being used by healthcare professionals to manage the symptoms and combat the impact of the virus on patients. Finally, this review provides an outline of the challenges and implications of CoVID-19 in a rural population, given that a large percentage of the population in a rural setting are frail subjects with pre-existing comorbidities, individuals with obesity, and individuals having a smoking history, who are at higher risk of mortality. Considering the age stratification, pre-existing co-morbidities such as obesity, smoking status along with limited healthcare access, resources, and health literacy, the rural population might be at a significant risk. Because of the dynamic nature of the pandemic, a conceptual framework is required for current and post-pandemic stages of CoVID-19, inclusive of strategies to mitigate the risks of this contagion in the rural population.

## References

[1] Cucinotta D., Vanelli M. (2020). WHO Declares COVID-19 a Pandemic. Acta Biomed..

[2] Fehr A.R., Perlman S. (2015). Coronaviruses: An overview of their replication and pathogenesis. Methods Mol. Biol..

[3] Cheng V.C., Lau S.K., Woo P.C., Yuen K.Y. (2007). Severe acute respiratory syndrome coronavirus as an agent of emerging and reemerging infection. Clin. Microbiol. Rev..

[4] Zhou P., Yang X.L., Wang X.G., Hu B., Zhang L., Zhang W., Si H.R., Zhu Y., Li B., Huang C.L. (2020). A pneumonia outbreak associated with a new coronavirus of probable bat origin. Nature.

[5] Sundaram N., Schaetti C., Purohit V., Kudale A., Weiss M.G. (2014). Cultural epidemiology of pandemic influenza in urban and rural Pune, India: A cross-sectional, mixed-methods study. BMJ Open.

[6] Richman L., Pearson J., Beasley C., Stanifer J. (2019). Addressing health inequalities in diverse, rural communities: An unmet need. SSM Popul. Health.

[7] Cosby A.G., McDoom-Echebiri M.M., James W., Khandekar H., Brown W., Hanna H.L. (2019). Growth and Persistence of Place-Based Mortality in the United States: The Rural Mortality Penalty. Am. J. Public Health.

[8] Zumla A., Chan J.F., Azhar E.I., Hui D.S., Yuen K.Y. (2016). Coronaviruses-drug discovery and therapeutic options. Nat. Rev. Drug Discov..

[9] Forni D., Cagliani R., Clerici M., Sironi M. (2017). Molecular Evolution of Human Coronavirus Genomes. Trends Microbiol..

[10] Kirchdoerfer R.N., Ward A.B. (2019). Structure of the SARS-CoV nsp12 polymerase bound to nsp7 and nsp8 co-factors. Nat. Commun..

[11] Li F., Li W., Farzan M., Harrison S.C. (2005). Structure of SARS coronavirus spike receptor-binding domain complexed with receptor. Science.

[12] Lu R., Zhao X., Li J., Niu P., Yang B., Wu H., Wang W., Song H., Huang B., Zhu N. (2020). Genomic characterisation and epidemiology of 2019 novel coronavirus: Implications for virus origins and receptor binding. Lancet.

[13] Lim Y.X., Ng Y.L., Tam J.P., Liu D.X. (2016). Human Coronaviruses: A Review of Virus-Host Interactions. Diseases.

[14] Yeager C.L., Ashmun R.A., Williams R.K., Cardellichio C.B., Shapiro L.H., Look A.T., Holmes K.V. (1992). Human aminopeptidase N is a receptor for human coronavirus 229E. Nature.

[15] Li W., Moore M.J., Vasilieva N., Sui J., Wong S.K., Berne M.A., Somasundaran M., Sullivan J.L., Luzuriaga K., Greenough T.C. (2003). Angiotensin-converting enzyme 2 is a functional receptor for the SARS coronavirus. Nature.

[16] Li W., Sui J., Huang I.C., Kuhn J.H., Radoshitzky S.R., Marasco W.A., Choe H., Farzan M. (2007). The S proteins of human coronavirus NL63 and severe acute respiratory syndrome coronavirus bind overlapping regions of ACE2. Virology.

[17] Wu K., Li W., Peng G., Li F. (2009). Crystal structure of NL63 respiratory coronavirus receptor-binding domain complexed with its human receptor. Proc. Natl. Acad. Sci. USA.

[18] van Doremalen N., Miazgowicz K.L., Milne-Price S., Bushmaker T., Robertson S., Scott D., Kinne J., McLellan J.S., Zhu J., Munster V.J. (2014). Host species restriction of Middle East respiratory syndrome coronavirus through its receptor, dipeptidyl peptidase 4. J. Virol..

[19] Huang X., Dong W., Milewska A., Golda A., Qi Y., Zhu Q.K., Marasco W.A., Baric R.S., Sims A.C., Pyrc K. (2015). Human Coronavirus HKU1 Spike Protein Uses O-Acetylated Sialic Acid as an Attachment Receptor Determinant and Employs Hemagglutinin-Esterase Protein as a Receptor-Destroying Enzyme. J. Virol..

[20] Butler N., Pewe L., Trandem K., Perlman S. (2006). Murine encephalitis caused by HCoV-OC43, a human coronavirus with broad species specificity, is partly immune-mediated. Virology.

[21] Wrapp D., Wang N., Corbett K.S., Goldsmith J.A., Hsieh C.L., Abiona O., Graham B.S., McLellan J.S. (2020). Cryo-EM structure of the 2019-nCoV spike in the prefusion conformation. Science.

[22] Perico L., Benigni A., Remuzzi G. (2020). Should COVID-19 Concern Nephrologists? Why and to What Extent? The Emerging Impasse of Angiotensin Blockade. Nephron.

[23] Ye M., Wysocki J., William J., Soler M.J., Cokic I., Batlle D. (2006). Glomerular localization and expression of Angiotensin-converting enzyme 2 and Angiotensin-converting enzyme: Implications for albuminuria in diabetes. J. Am. Soc. Nephrol..

[24] Shereen M.A., Khan S., Kazmi A., Bashir N., Siddique R. (2020). COVID-19 infection: Origin, transmission, and characteristics of human coronaviruses. J. Adv. Res..

[25] Mackenzie J.S., Smith D.W. (2020). COVID-19: A novel zoonotic disease caused by a coronavirus from China: What we know and what we don’t. Microbiol Aust..

[26] Li H., Liu S.M., Yu X.H., Tang S.L., Tang C.K. (2020). Coronavirus disease 2019 (COVID-19): Current status and future perspectives. Int. J. Antimicrob. Agents.

[27] Backer J.A., Klinkenberg D., Wallinga J. (2020). Incubation period of 2019 novel coronavirus (2019-nCoV) infections among travellers from Wuhan, China, 20-28 January 2020. Euro Surveill..

[28] Lauer S.A., Grantz K.H., Bi Q., Jones F.K., Zheng Q., Meredith H.R., Azman A.S., Reich N.G., Lessler J. (2020). The Incubation Period of Coronavirus Disease 2019 (COVID-19) From Publicly Reported Confirmed Cases: Estimation and Application. Ann. Intern. Med..

[29] Singhal T. (2020). A Review of Coronavirus Disease-2019 (COVID-19). Indian. J. Pediatr..

[30] Zou L., Ruan F., Huang M., Liang L., Huang H., Hong Z., Yu J., Kang M., Song Y., Xia J. (2020). SARS-CoV-2 Viral Load in Upper Respiratory Specimens of Infected Patients. N. Engl. J. Med..

[31] Pan Y., Zhang D., Yang P., Poon L.L.M., Wang Q. (2020). Viral load of SARS-CoV-2 in clinical samples. Lancet Infect. Dis..

[32] Qu G., Li X., Hu L., Jiang G. (2020). An Imperative Need for Research on the Role of Environmental Factors in Transmission of Novel Coronavirus (COVID-19). Environ. Sci Technol..

[33] Weber D.J., Rutala W.A., Fischer W.A., Kanamori H., Sickbert-Bennett E.E. (2016). Emerging infectious diseases: Focus on infection control issues for novel coronaviruses (Severe Acute Respiratory Syndrome-CoV and Middle East Respiratory Syndrome-CoV), hemorrhagic fever viruses (Lassa and Ebola), and highly pathogenic avian influenza viruses, A(H5N1) and A(H7N9). Am. J. Infect. Control..

[34] Moriyama M., Hugentobler W.J., Iwasaki A. (2020). Seasonality of Respiratory Viral Infections. Annu. Rev. Virol..

[35] Suman R., Javaid M., Haleem A., Vaishya R., Bahl S., Nandan D. (2020). Sustainability of Coronavirus on different surfaces. J. Clin. Exp. Hepatol..

[36] Casanova L., Rutala W.A., Weber D.J., Sobsey M.D. (2009). Survival of surrogate coronaviruses in water. Water Res..

[37] Li Q., Guan X., Wu P., Wang X., Zhou L., Tong Y., Ren R., Leung K.S.M., Lau E.H.Y., Wong J.Y. (2020). Transmission Dynamics in Wuhan, China, of Novel Coronavirus-Infected Pneumonia. N. Engl. J. Med..

[38] Cheng Z.J., Shan J. (2020). 2019 Novel coronavirus: Where we are and what we know. Infection.

[39] Zhao S., Lin Q., Ran J., Musa S.S., Yang G., Wang W., Lou Y., Gao D., Yang L., He D. (2020). Preliminary estimation of the basic reproduction number of novel coronavirus (2019-nCoV) in China, from 2019 to 2020: A data-driven analysis in the early phase of the outbreak. Int. J. Infect. Dis..

[40] Zhai P., Ding Y., Wu X., Long J., Zhong Y., Li Y. (2020). The epidemiology, diagnosis and treatment of COVID-19. Int J. Antimicrob Agents.

[41] Cascella M., Rajnik M., Cuomo A., Dulebohn S.C., Di Napoli R. (2020). Features, Evaluation and Treatment Coronavirus (COVID-19). StatPearls.

[42] Guo Y.R., Cao Q.D., Hong Z.S., Tan Y.Y., Chen S.D., Jin H.J., Tan K.S., Wang D.Y., Yan Y. (2020). The origin, transmission and clinical therapies on coronavirus disease 2019 (COVID-19) outbreak-an update on the status. Mil. Med. Res..

[43] Chen N., Zhou M., Dong X., Qu J., Gong F., Han Y., Qiu Y., Wang J., Liu Y., Wei Y. (2020). Epidemiological and clinical characteristics of 99 cases of 2019 novel coronavirus pneumonia in Wuhan, China: A descriptive study. Lancet.

[44] Meo S.A., Alhowikan A.M., Al-Khlaiwi T., Meo I.M., Halepoto D.M., Iqbal M., Usmani A.M., Hajjar W., Ahmed N. (2020). Novel coronavirus 2019-nCoV: Prevalence, biological and clinical characteristics comparison with SARS-CoV and MERS-CoV. Eur. Rev. Med. Pharmacol. Sci..

[45] Adhikari S.P., Meng S., Wu Y.J., Mao Y.P., Ye R.X., Wang Q.Z., Sun C., Sylvia S., Rozelle S., Raat H. (2020). Epidemiology, causes, clinical manifestation and diagnosis, prevention and control of coronavirus disease (COVID-19) during the early outbreak period: A scoping review. Infect. Dis. Poverty.

[46] Holshue M.L., DeBolt C., Lindquist S., Lofy K.H., Wiesman J., Bruce H., Spitters C., Ericson K., Wilkerson S., Tural A. (2020). Washington State -nCo, VCIT: First Case of 2019 Novel Coronavirus in the United States. N. Engl. J. Med..

[47] Huang C., Wang Y., Li X., Ren L., Zhao J., Hu Y., Zhang L., Fan G., Xu J., Gu X. (2020). Clinical features of patients infected with 2019 novel coronavirus in Wuhan, China. Lancet.

[48] Wang Y., Wang Y., Chen Y., Qin Q. (2020). Unique epidemiological and clinical features of the emerging 2019 novel coronavirus pneumonia (COVID-19) implicate special control measures. J. Med. Virol..

[49] Guan W.J., Ni Z.Y., Hu Y., Liang W.H., Ou C.Q., He J.X., Liu L., Shan H., Lei C.L., Hui D.S.C. (2020). China Medical Treatment Expert Group for, C. Clinical Characteristics of Coronavirus Disease 2019 in China. N. Engl J. Med..

[50] Wu Z., McGoogan J.M. (2020). Characteristics of and Important Lessons From the Coronavirus Disease 2019 (COVID-19) Outbreak in China: Summary of a Report of 72314 Cases From the Chinese Center for Disease Control and Prevention. JAMA.

[51] Chan J.F., Yuan S., Kok K.H., To K.K., Chu H., Yang J., Xing F., Liu J., Yip C.C., Poon R.W. (2020). A familial cluster of pneumonia associated with the 2019 novel coronavirus indicating person-to-person transmission: A study of a family cluster. Lancet.

[52] Gao M., Yang L., Chen X., Deng Y., Yang S., Xu H., Chen Z., Gao X. (2020). A study on infectivity of asymptomatic SARS-CoV-2 carriers. Respir. Med..

[53] Weisgrau S. (1995). Issues in rural health: Access, hospitals, and reform. Health Care Financ Rev..

[54] Trivedi T., Liu J., Probst J., Merchant A., Jhones S., Martin A.B. (2015). Obesity and obesity-related behaviors among rural and urban adults in the USA. Rural Remote Health.

[55] Guan W.J., Liang W.H., Zhao Y., Liang H.R., Chen Z.S., Li Y.M., Liu X.Q., Chen R.C., Tang C.L., Wang T. (2020). China Medical Treatment Expert Group for, C. Comorbidity and its impact on 1590 patients with COVID-19 in China: A nationwide analysis. Eur. Respir. J..

[56] Wang B., Li R., Lu Z., Huang Y. (2020). Does comorbidity increase the risk of patients with COVID-19: Evidence from meta-analysis. Aging (Albany NY).

[57] Ranscombe P. (2020). Rural areas at risk during COVID-19 pandemic. Lancet. Infect. Dis..

[58] Cheng P., Zhu H., Witteles R.M., Wu J.C., Quertermous T., Wu S.M., Rhee J.W. (2020). Cardiovascular Risks in Patients with COVID-19: Potential Mechanisms and Areas of Uncertainty. Curr. Cardiol. Rep..

[59] Marhl M., Grubelnik V., Magdic M., Markovic R. (2020). Diabetes and metabolic syndrome as risk factors for COVID-19. Diabetes Metab. Syndr..

[60] Sheahan T.P., Sims A.C., Graham R.L., Menachery V.D., Gralinski L.E., Case J.B., Leist S.R., Pyrc K., Feng J.Y., Trantcheva I. (2017). Broad-spectrum antiviral GS-5734 inhibits both epidemic and zoonotic coronaviruses. Sci. Transl. Med..

[61] Mulangu S., Dodd L.E., Davey R.T., Tshiani Mbaya O., Proschan M., Mukadi D., Lusakibanza Manzo M., Nzolo D., Tshomba Oloma A., Ibanda A. (2019). A Randomized, Controlled Trial of Ebola Virus Disease Therapeutics. N. Engl. J. Med..

[62] Vincent M.J., Bergeron E., Benjannet S., Erickson B.R., Rollin P.E., Ksiazek T.G., Seidah N.G., Nichol S.T. (2005). Chloroquine is a potent inhibitor of SARS coronavirus infection and spread. Virol. J..

[63] Ruiz-Irastorza G., Ramos-Casals M., Brito-Zeron P., Khamashta M.A. (2010). Clinical efficacy and side effects of antimalarials in systemic lupus erythematosus: A systematic review. Ann. Rheum. Dis..

[64] van den Borne B.E., Dijkmans B.A., de Rooij H.H., le Cessie S., Verweij C.L. (1997). Chloroquine and hydroxychloroquine equally affect tumor necrosis factor-alpha, interleukin 6, and interferon-gamma production by peripheral blood mononuclear cells. J. Rheumatol..

[65] Stockman L.J., Bellamy R., Garner P. (2006). SARS: Systematic review of treatment effects. PLoS Med..

[66] Chu C.M., Cheng V.C., Hung I.F., Wong M.M., Chan K.H., Chan K.S., Kao R.Y., Poon L.L., Wong C.L., Guan Y. (2004). Group, HUSS. Role of lopinavir/ritonavir in the treatment of SARS: Initial virological and clinical findings. Thorax.

[67] Delang L., Abdelnabi R., Neyts J. (2018). Favipiravir as a potential countermeasure against neglected and emerging RNA viruses. Antiviral. Res..

[68] Hoffmann M., Kleine-Weber H., Schroeder S., Kruger N., Herrler T., Erichsen S., Schiergens T.S., Herrler G., Wu N.H., Nitsche A. (2020). SARS-CoV-2 Cell Entry Depends on ACE2 and TMPRSS2 and Is Blocked by a Clinically Proven Protease Inhibitor. Cell.

[69] Coleman C.M., Sisk J.M., Mingo R.M., Nelson E.A., White J.M., Frieman M.B. (2016). Abelson Kinase Inhibitors Are Potent Inhibitors of Severe Acute Respiratory Syndrome Coronavirus and Middle East Respiratory Syndrome Coronavirus Fusion. J. Virol..

[70] Sun M.L., Yang J.M., Sun Y.P., Su G.H. (2020). Inhibitors of RAS Might Be a Good Choice for the Therapy of COVID-19 Pneumonia. Zhonghua Jie He He Hu Xi Za Zhi.

[71] Sodhi K., Wu C.C., Cheng J., Gotlinger K., Inoue K., Goli M., Falck J.R., Abraham N.G., Schwartzman M.L. (2010). CYP4A2-induced hypertension is 20-hydroxyeicosatetraenoic acid- and angiotensin II-dependent. Hypertension.

[72] Zhang L., Liu Y. (2020). Potential interventions for novel coronavirus in China: A systematic review. J. Med. Virol..

[73] Mousa H.A. (2017). Prevention and Treatment of Influenza, Influenza-Like Illness, and Common Cold by Herbal, Complementary, and Natural Therapies. J. Evid. Based Complementary Altern. Med..

[74] Shi Y., Wang Y., Shao C., Huang J., Gan J., Huang X., Bucci E., Piacentini M., Ippolito G., Melino G. (2020). COVID-19 infection: The perspectives on immune responses. Cell Death Differ..

[75] Ren J.L., Zhang A.H., Wang X.J. (2020). Traditional Chinese medicine for COVID-19 treatment. Pharmacol. Res..

[76] Ang L., Lee H.W., Choi J.Y., Zhang J., Soo Lee M. (2020). Herbal medicine and pattern identification for treating COVID-19: A rapid review of guidelines. Integr. Med. Res..

[77] Ang L., Song E., Lee H.W., Lee M.S. (2020). Herbal Medicine for the Treatment of Coronavirus Disease 2019 (COVID-19): A Systematic Review and Meta-Analysis of Randomized Controlled Trials. J. Clin. Med..

[78] (2020). Redeploying plant defences. Nat. Plants.

[79] Krishna S., Bustamante L., Haynes R.K., Staines H.M. (2008). Artemisinins: Their growing importance in medicine. Trends Pharmacol. Sci..

[80] Yang Y., Islam M.S., Wang J., Li Y., Chen X. (2020). Traditional Chinese Medicine in the Treatment of Patients Infected with 2019-New Coronavirus (SARS-CoV-2): A Review and Perspective. Int. J. Biol. Sci..

[81] Yang C., Hu D.H., Feng Y. (2015). Essential oil of Artemisia vestita exhibits potent in vitro and in vivo antibacterial activity: Investigation of the effect of oil on biofilm formation, leakage of potassium ions and survival curve measurement. Mol. Med. Rep..

[82] Team C.C.-R. (2020). Severe Outcomes Among Patients with Coronavirus Disease 2019 (COVID-19)-United States, February 12–March 16, 2020. MMWR Morb. Mortal Wkly. Rep..

[83] Wong H., Moore K., Angstman K.B., Garrison G.M. (2019). Impact of rural address and distance from clinic on depression outcomes within a primary care medical home practice. BMC Fam. Pract..

[84] Dietz W., Santos-Burgoa C. (2020). Obesity and its Implications for COVID-19 Mortality. Obesity (Silver Spring).

[85] Doogan N.J., Roberts M.E., Wewers M.E., Stanton C.A., Keith D.R., Gaalema D.E., Kurti A.N., Redner R., Cepeda-Benito A., Bunn J.Y. (2017). A growing geographic disparity: Rural and urban cigarette smoking trends in the United States. Prev. Med..

[86] Scoglio C., Schumm W., Schumm P., Easton T., Roy Chowdhury S., Sydney A., Youssef M. (2010). Efficient mitigation strategies for epidemics in rural regions. PLoS ONE.

[87] Kirakli C., Tatar D., Cimen P., Edipoglu O., Coskun M., Celikten E., Ozsoz A. (2011). Survival from severe pandemic H1N1 in urban and rural Turkey: A case series. Respir. Care.

[88] Wallace D.J., Seymour C.W., Kahn J.M. (2017). Hospital-Level Changes in Adult ICU Bed Supply in the United States. Crit. Care Med..

[89] Ricketts T.C. (2000). Health care in rural communities. West. J. Med..

[90] Nielsen M., D’Agostino D., Gregory P. (2017). Addressing Rural Health Challenges Head On. Mo. Med..

[91] Moser D.K., Robinson S., Biddle M.J., Pelter M.M., Nesbitt T.S., Southard J., Cooper L., Dracup K. (2015). Health Literacy Predicts Morbidity and Mortality in Rural Patients With Heart Failure. J. Card. Fail..

[92] Parker R.M., Wolf M.S., Kirsch I. (2008). Preparing for an epidemic of limited health literacy: Weathering the perfect storm. J. Gen. Intern. Med..

[93] Dewalt D.A., Berkman N.D., Sheridan S., Lohr K.N., Pignone M.P. (2004). Literacy and health outcomes: A systematic review of the literature. J. Gen. Intern. Med..

[94] Wolf M.S., Gazmararian J.A., Baker D.W. (2005). Health literacy and functional health status among older adults. Arch. Intern. Med..

[95] Vaughan E., Tinker T. (2009). Effective health risk communication about pandemic influenza for vulnerable populations. Am. J. Public Health.

[96] Ramirez A.S., Estrada E., Ruiz A. (2017). Mapping the Health Information Landscape in a Rural, Culturally Diverse Region: Implications for Interventions to Reduce Information Inequality. J. Prim. Prev..

